# Temporal Stability of Epigenetic Markers: Sequence Characteristics and Predictors of Short-Term DNA Methylation Variations

**DOI:** 10.1371/journal.pone.0039220

**Published:** 2012-06-20

**Authors:** Hyang-Min Byun, Francesco Nordio, Brent A. Coull, Letizia Tarantini, Lifang Hou, Matteo Bonzini, Pietro Apostoli, Pier Alberto Bertazzi, Andrea Baccarelli

**Affiliations:** 1 Laboratory of Environmental Epigenetics, Exposure Epidemiology and Risk Program, Harvard School of Public Health, Boston, Massachusetts, United States of America; 2 Department of Environmental and Occupational Health, Università degli Studi di Milano and IRCCS Ca’ Granda Maggiore Policlinico Hospital, Milan, Italy; 3 Department of Biostatistics, Harvard School of Public Health, Boston, Massachusetts, United States of America; 4 Department of Preventive Medicine, Feinberg School of Medicine, Northwestern University, Chicago, Illinois, United States of America; 5 Epidemiology and Preventive Medicine Research Centre, University of Insubria, Varese, Italy; 6 Department of Experimental and Applied Medicine, Occupational Medicine and Industrial Hygiene, University of Brescia, Brescia, Italy; Geisel School of Medicine at Dartmouth, United States of America

## Abstract

**Background:**

DNA methylation is an epigenetic mechanism that has been increasingly investigated in observational human studies, particularly on blood leukocyte DNA. Characterizing the degree and determinants of DNA methylation stability can provide critical information for the design and conduction of human epigenetic studies.

**Methods:**

We measured DNA methylation in 12 gene-promoter regions (*APC, p16, p53, RASSF1A, CDH13, eNOS, ET-1, IFNγ, IL-6, TNFα, iNOS,* and *hTERT*) and 2 of non-long terminal repeat elements, i.e., L1 and *Alu* in blood samples obtained from 63 healthy individuals at baseline (Day 1) and after three days (Day 4). DNA methylation was measured by bisulfite-PCR-Pyrosequencing. We calculated intraclass correlation coefficients (ICCs) to measure the within-individual stability of DNA methylation between Day 1 and 4, subtracted of pyrosequencing error and adjusted for multiple covariates.

**Results:**

Methylation markers showed different temporal behaviors ranging from high (*IL-6*, ICC = 0.89) to low stability (*APC*, ICC = 0.08) between Day 1 and 4. Multiple sequence and marker characteristics were associated with the degree of variation. Density of CpG dinucleotides nearby the sequence analyzed (measured as CpG(o/e) or G+C content within ±200bp) was positively associated with DNA methylation stability. The 3′ proximity to repeat elements and range of DNA methylation on Day 1 were also positively associated with methylation stability. An inverted U-shaped correlation was observed between mean DNA methylation on Day 1 and stability.

**Conclusions:**

The degree of short-term DNA methylation stability is marker-dependent and associated with sequence characteristics and methylation levels.

## Introduction

DNA methylation is a well-studied epigenetic mechanism that has been increasingly investigated in epidemiology studies in relation to a variety of risk factors and health-related conditions, including aging, prenatal, early- and adult-life risk factors, changing environments, and disease outcomes. Albeit DNA methylation shows dynamic changes during developmental stages, DNA methylation markings have been suggested to be relatively stable over time in adult individuals [1]. DNA methylation changes can be replicated through cell mitosis and persist even in the absence of the conditions that established them [1]. Due to its stability, DNA methylation has been suggested to be particularly well suited to represent ‘the interactions of genes with their environment, which bring the phenotype onto being’ [1], and to ‘record a variety of dietary, lifestyle, behavioral, and social cues’ [2].

Experimental models and human studies, however, indicate that DNA methylation can exhibit different temporal behaviors, varying between the nearly absolute stability of the DNA sequence and the rapid variations typical of mRNA levels. DNA methylation in imprinted genes, for instance, is established in early embryogenesis and is believed to remain relatively stable throughout the life-course [3]. Some non-imprinted genes, as well as non-coding repeat elements, have been suggested to undergo slow progressive changes in DNA methylation through aging [4]. On the other hand, some other non-imprinted sequences – such as the *IL-2* promoter – have been demonstrated to undergo profound changes in DNA methylation as rapidly as within 20 minutes after an exogenous challenge [5]. However, no *in-vivo* human data are yet available to help distinguish sequences that undergo rapid changes in DNA methylation from those with more stable methylation levels. This information would be relevant for effective design of epidemiology studies, as well as for statistical analysis of methylation data. In studies of risk factors that operate over a short timeframe, such as investigations on triggers of cardiovascular events including air pollution, alcohol, and cocaine abuse [6], [7], investigators may be interested in focusing on epigenetic markers that vary their methylation levels over a relatively short timeframe. Conversely, longitudinal studies evaluating effects of cumulative risk factor exposures and associations with risk of chronic diseases would best avoid expending resources and statistical power on sequences with rapid DNA variation.

The International Human Epigenome Consortium is expected to map 1,000 reference epigenomes and define the level of variation that exists between individuals and across different tissues [8]. However, no information is available on the dynamics of DNA methylation changes in normal tissues and to identify genomic characteristics that may be associated with rapid variations in methylation changes. In various studies, DNA methylation shows genomic region specific changes in terms of CpG density. CpG-rich regions (generally called CpG islands) are commonly unmethylated in normal human tissues and have been found to show DNA methylation changes in normal healthy individuals through aging [9]. CpG-island shores – i.e., areas with low CpG density bordering CpG-rich regions known as CpG islands - show frequent variations of DNA methylation between cancer and normal tissues, as well as in stem cells compared with differentiated tissues [10]. On the other hand, a low CpG density area, distinct from shores or CpG islands, was identified as a primary region for the transgenerational differentially methylated regions (DMR) [11]. In vitro data show that, transcription complexes, such as transcription factors, nucleosome occupancy, chromatin contents [12], [13], show differential binding preference to genomic regions depending on CpG density. DNA methyltransferases (DNMTs) interactions with the transcription machinery [14] may bring DNA methylation variation dependent on CpG density. Also, proximity to repeat elements is another genomic feature that might affect DNA methylation stability. For instance, Alu repeat elements have been proposed as methylation centers that can contribute to propagate DNA methylation to nearby gene promoter CpGs [15], [16], [17].

Blood DNA from unfractionated peripheral leukocytes has been most frequently used in DNA methylation analyses in epidemiology studies [18], as it can be easily obtained from living human individuals and used for DNA methylation analysis following standard collection and isolation techniques. Due to the interest in the investigation of genetic and molecular markers, a wealth of epidemiology studies have collected and stored blood leukocyte DNA, which can be readily used for DNA methylation analyses. Peripheral blood leukocytes have been suggested to directly or indirectly participate in the pathophysiology of a wide array of human diseases that are initiated by or associated with systemic inflammatory and immune responses, including – but not limited to – immune, infectious, cardiovascular, and respiratory disease [19]. As a result, DNA methylation analyses on blood DNA have been conducted in investigations of ischemic heart disease [20], [21], stroke [21], autoimmune connective-tissue disease [22], as well as of psychiatric disease [23], neurological disorders [24], and various cancers [25].

In the present study, we report analyses on DNA methylation markers with different genomic characteristics aimed at: i) characterizing short-term variability in blood DNA methylation; and ii) identifying characteristics associated with the variability in DNA methylation levels. We investigated a population exposed to an environmental risk factor, i.e., metal-rich airborne particulate matter (PM), which has been previously associated with short-term variations in DNA methylation [26]. We used statistical methods accounting for measurement error from pyrosequencing analyses, as well as potential effects on DNA methylation from modifications in the proportion of leukocyte cell types. We show examples of markers with rapid variation in DNA methylation, and we demonstrate sequence and other marker characteristics that are associated with DNA methylation stability.

## Materials and Methods

### Study Participants

The present study is based on 63 male healthy workers of a Northern-Italy electric steel plant, free of cancer, cardiovascular, and pulmonary disease [26], [27]. Characteristics and exposure levels of the study participants were reported previously [26]. An in-person interview collected detailed individual and lifestyle information. For each participant, we obtained blood samples on two different days: on the first day of a workweek (Day 1); and after three days of work (Day 4). This study was approved by Università degli Studi di Milano and IRCCS Ca’ Granda Maggiore Policlinico Hospital and all samples were collected according to the institutional review board of the Università degli Studi di Milano and IRCCS Ca’ Granda Maggiore Policlinico Hospital in accordance with institutional guidelines. Individual written informed consent was obtained from all participants before the study.

### Blood Sample Collection and DNA Methylation Analyses

Day 1 and 4 blood samples were collected and processed using the same protocols, as previously described [26]. After purification, Day 1 and Day 4 DNA samples were interspersed across plates to minimize plate effect bias. We measured DNA methylation in 12 genes [Adenomatous polyposis coli (*APC*), cyclin-dependent kinase 2a (*p16*), tumor-protein p53 (*p53*), Ras-association (RalGDS/AF-6) domain family member 1 (*RASSF1A*), cadherin 13 (*CDH13*), nitric oxide synthase 3 (*eNOS*), endothelin-1 (*ET-1*), interferon, gamma (*IFNγ*), interleukin-6 (*IL-6*), tumor-necrosis factor α (*TNFα*), nitric-oxide synthase 2, inducible (*iNOS*), and telomerase reverse transcriptase (*hTERT)*]; and two repeat elements (L1 [also known as LINE-1] and *Alu*). All methylation markers were selected as candidates for their potential participation in pathway activated by PM exposures. Bisulfite-PCR-pyrosequencing was performed as previously described [26]. PCR primers and PCR conditions for promoter regions of genes and repeat elements are listed in Table S1. All post-PCR products were run twice on pyrosequencing to increase precision.

### Characteristics of the DNA Methylation Markers

In order to study the correlation between sequence characteristics and stability of each methylation marker, we determined the following characteristics: GC content (G+C) and ratio of observed-to-expected CpG [CpG(o/e)] at ±200bp from the first CpG site evaluated; and distance of any repeat elements from target CpGs in 5′ or 3′ direction (Table 1). GC content and CpG(o/e) data were obtained using the web-based program *cpgplot* (http://www.ebi.ac.uk/Tools/emboss/cpgplot/#andNewcpgseek). The distance of repeat elements from target CpGs was calculated manually using the UCSC web browser (http://genome.ucsc.edu/). We used information on ten classes of repeats, included short and long interspersed nuclear elements (SINEs and LINEs), long terminal repeat elements (LTRs), DNA repeat elements, simple repeats (micro-satellites), low-complexity repeats, satellite repeats, RNA repeats (including RNA, tRNA, rRNA, snRNA, scRNA, srpRNA), and other repeats (including repeats classified as class RC [Rolling Circle] and ‘unknown’).

**Table 1:** Sequence characteristics of the DNA methylation markers analyzed.

Gene	G+C	CpGo/e	Repeat elements: distance at 3'	Repeat elements: distance at 5'	# CpG positions analyzed
***APC***	0.70	0.72	1847	371	4
***CDH13***	0.39	0.27	0.00	0.00	2
***eNOS***	0.66	0.29	592	1411	3
***ET-1***	0.60	0.86	2570	2264	4
***hTERT***	0.70	0.54	873	3466	3
***IFNγ***	0.39	0.39	874	934	2
***IL-6***	0.59	0.59	460	1503	2
***iNOS***	0.56	0.19	1004	520	2
***p16***	0.72	0.74	699	1093	7
***p53***	0.57	0.56	1484	408	4
***RASSF1A***	0.64	0.69	1440	3208	4
***TNFα***	0.57	0.50	568	1327	4
***Alu***	–*	–*	–*	–*	3
**LINE-1**	–*	–*	–*	–*	3

* *Alu* and LINE-1 were not considered, as repeat elements have multiple locations across the human genome with different context sequence characteristics.

### Statistical Analysis

To account for the data structure, we used the following random-effect model:
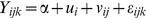
(1)where Y_ijk_ represents the methylation value for individual *i* = 1,2,…,63, on Day *j = 1,2,* for technical pyrosequencing replicate *k = 1,2*; α is an overall intercept, and u_i_ and v_ij_ are random effects associated with individual and day within individual; ε_ijk_ is the error term. As is standard in linear mixed effects models, we assume the random effects and residual errors are normally distributed: u_i_∼N (0, σ_ID_); v_ij_∼N (0, σ_ID, Day_); and ε_ijk_∼N (0, σ_Run_), yielding three variance components (σ_ID_, σ_ID, Day_, σ_Run_) in the model. The residual variance σ_Run_ captures the pyrosequencing measurement error measured by the technical replicates (pyrosequencing runs) on the same individual and day. We used intraclass correlation coefficients (ICCs) to estimate DNA methylation stability. We expected the ICCs to vary between 0 (i.e., no correlation between Day 1 and 4) and 1 (i.e., maximum stability between Day 1 and 4). Define σ_TOT_ = σ_ID_+σ_ID, Day_+σ_Run_. We calculated two versions of the ICC, ICC_1_ and ICC_2,_ using the following formulas:



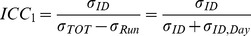
(2)

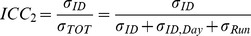
(3)


ICC_1_ was calculated to estimate within-individual DNA methylation stability, excluding pyrosequencing measurement error (σ_Run_). ICC_2_ includes all three sources of variability the denominator. We fitted unadjusted models, as well as models adjusted for PM_10_ exposure levels, age, current smoking, and percent blood granulocytes. Percent blood granulocytes are the most represented cell type among nucleated blood cells and were therefore included in the adjusted models as an independent variable to account for potential differences in DNA methylation due to between-day changes in the proportions of leukocyte subtypes. As a sensitivity analysis, we also added to the adjusted models percent lymphocytes and percent monocytes. ICCs from this sensitivity analysis (Table S2) did not show any major differences from those calculated from the models adjusted only for PM_10_, age, current smoking, and percent blood granulocytes.

To evaluate the potential determinants of the differences in DNA methylation stability between the markers, we fitted simple linear regression models in which the dependent variable was ICC and the independent variable was one of the characteristics of the methylation markers (i.e., G+C density, CpG(o/e), distance from repeat elements at 3′, distance from repeat elements at 5′; mean methylation value, or range of methylation). Covariate-adjusted ICC_1_, as calculated above, were used in this set of analyses. In addition to the linear models, we also tested non-linear relationships between ICC and each of the marker characteristics via regression models including a quadratic term. As sensitivity analysis, we fit the same regression models after logit transformation of ICC, because ICC is a proportion ranged between 0 and 1 and non-normal distributed. Results from this set of sensitivity analysis (shown in Table S3 and Figure S1) did not show major departures from the results of the primary analysis. All statistical analyses were performed in SAS (version 9.2; SAS Institute Inc., Cary, NC, USA).

## Results

### Levels and Characteristics of the DNA Methylation Markers

We measured DNA methylation in 12 gene promoter regions (*APC, p16, p53, RASSF1A, CDH13, eNOS, ET-1, IFNγ, IL-6, TNFα, iNOS,* and *hTERT*) and 2 of non-long terminal repeat elements, i.e., LINE-1 and *Alu* (Table 1). The markers showed extensive differences in the proportion of guanosine and cytosine content (G+C), which varied between 0.39 (*IFNγ*) and 0.72 (*p16*). The ratio of observed over expected CpG frequency [CpG(o/e)] varied between 0.19 (*iNOS*) and 0.86 (*ET-1*). The markers also had large differences in the distances from the nearest repeat element in 5′ (ranging between 0 bp [*CDH13*] and 3.5 kB [*hTERT*]) and 3′ (ranging between 0 bp [*CDH13*] and 2.6 kB [*ET-1*]). In each marker, the assays we designed allowed for measuring a variable number of CpG sites, ranging from 2 to 7. The number of CpGs was dependent on the CpG density in the vicinity of the target area, as we were able to analyze a higher number of CpGs in sequences with higher CpG density. In the statistical analysis, we used the average of the CpG sites in each marker. We also conducted sensitivity analyses using the methylation values at each of the CpG sites within each marker, as shown below.

### Mean DNA Methylation Difference between Day 1 and 4

*APC*, *p16*, and *CDH13* showed small mean differences in DNA methylation levels between Day 1 and 4 (Table 2). *APC* methylation increased from 4.7% (SE = 0.13) to 4.9% (SE = 0.13%) (difference = 0.2, 95% CI = 0.04; 0.44). *p16* methylation increased from 2.2% (SE = 0.09) to 2.4% (SE = 0.09) (difference = 0.2, 95% CI = 0.04; 0.3). *CDH13* methylation decreased from 78.0% (SE = 0.33) to 77.4% (SE = 0.35) (difference = −0.6, 95% CI = −1.1; −0.06). *iNOS* methylation decreased from 68.2% (SE = 0.46) to 67.6% (SE = 0.48) (difference = −0.6, 95% CI = −1.2; −0.02). All the other markers did not show clear differences in DNA methylation between the two time points. The analyses on individual CpGs were overall similar to the analysis based on the mean of CpGs (Table S4).

**Table 2:** Blood DNA methylation levels (%mC) in Day 1 and Day 4 samples.

Gene	Day 1		Day 4		Difference	(95% CI)
	Mean	(SE)	Mean	(SE)		
***APC***	4.7	(0.13)	4.9	(0.13)	0.2	(0.04; 0.4)
***CDH13***	78.0	(0.33)	77.4	(0.35)	−0.6	(−1.1; −0.06)
***eNOS***	91.9	(0.30)	92.0	(0.25)	0.1	(−0.3; 0.6)
***ET-1***	6.2	(0.42)	6.3	(0.40)	0.1	(−0.4; 0.6)
***hTERT***	92.6	(0.18)	92.6	(0.15)	0.0	(−0.3; 0.4)
***IFN*** **γ**	73.8	(0.78)	73.0	(0.73)	−0.8	(−2.0; 0.4)
***IL-6***	42.6	(0.65)	42.6	(0.62)	−0.0	(−0.6; 0.6)
***iNOS***	68.2	(0.46)	67.6	(0.48)	−0.6	(−1.2; −0.02)
***p16***	2.2	(0.09)	2.4	(0.09)	0.2	(0.04; 0.3)
***p53***	6.2	(0.17)	6.3	(0.17)	0.1	(−0.2; 0.3)
***RASSF1A***	7.5	(0.46)	7.1	(0.46)	−0.4	(−1.0; 0.2)
***TNF*** **α**	12.8	(0.33)	12.5	(0.33)	−0.3	(−0.8; 0.2)
***Alu***	25.8	(0.10)	25.8	(0.08)	−0.0	(−0.2; 0.2)
**LINE-1**	78.8	(0.13)	78.8	(0.15)	−0.0	(−0.4; 0.2)

### Stability of DNA Methylation Markers

We used ICCs to describe the stability of the DNA methylation markers between Day 1 and 4. ICCs capture both the mean and within-individual differences of the individual data points, thus providing an overall measure of biomarker stability. We decomposed the total variance in three components (Table 3), i.e., σ_ID,_ representing the between-individual variance in DNA methylation; σ_ID, Day_, representing the within-individual variance due to changes in DNA methylation between Day 1 and 4; and σ_Run_, representing the variance between duplicate pyrosequencing runs on the same sample (i.e., pyrosequencing error).

**Table 3:** Variance components and ICCs estimating the concordance between Day 1 and Day 4 DNA methylation measures.

	Unadjusted Models						Models adjusted by PM_10_ exposure levels, age, current smoking, and percent blood granulocytes					
Marker		σ_ID_	σ_ID, Day_	σ_Run_	ICC_1_	ICC_2_		σ_ID_	σ_ID, Day_	σ_Run_	ICC_1_	ICC_2_
***APC***		0.15	1.28	0.36	**0.10**	**0.08**		0.14	1.29	0.36	**0.10**	**0.08**
***CDH13***		4.98	2.36	0.25	**0.68**	**0.66**		4.72	2.26	0.25	**0.68**	**0.65**
***eNOS***		2.77	1.34	0.60	**0.67**	**0.59**		2.69	1.35	0.60	**0.67**	**0.58**
***ET-1***		4.92	7.30	0.13	**0.40**	**0.40**		5.37	7.33	0.13	**0.42**	**0.42**
***hTERT***		0.47	1.11	0.54	**0.30**	**0.22**		0.54	1.09	0.54	**0.33**	**0.25**
***IFNγ***		23.18	11.18	0.23	**0.67**	**0.67**		18.27	9.94	0.23	**0.65**	**0.64**
***IL-6***		22.07	2.68	0.30	**0.89**	**0.88**		22.74	2.65	0.30	**0.90**	**0.89**
***iNOS***		11.28	2.52	0.30	**0.82**	**0.80**		11.53	2.54	0.30	**0.82**	**0.80**
***p16***		0.15	0.49	0.07	**0.23**	**0.21**		0.16	0.49	0.07	**0.25**	**0.23**
***p53***		0.53	1.65	0.23	**0.24**	**0.22**		0.58	1.64	0.23	**0.26**	**0.24**
***RASSF1A***		7.41	11.13	0.15	**0.40**	**0.40**		8.15	11.00	0.15	**0.43**	**0.42**
***TNFα***		4.40	1.69	0.17	**0.72**	**0.70**		3.67	1.70	0.17	**0.68**	**0.66**
***Alu***		0.12	0.20	0.27	**0.39**	**0.21**		0.11	0.20	0.27	**0.37**	**0.20**
**LINE-1**		0.59	0.98	0.33	**0.38**	**0.31**		0.59	0.96	0.33	**0.38**	**0.31**

Annotation: σ_ID_ represents the between-subject variance in DNA methylation; σ_ID, Day_ represents the variance due to within-subject changes in DNA methylation between Day 1 and Day 4*;* σ_Run_ represents the variance between duplicate pyrosequencing runs on the same sample (i.e., analytical measurement error from pyrosequencing). Two types of Intraclass Correlation Coefficients (ICCs) were computed using the quantities above: ICC_1_, subtracted of the measurement error (σ_Run_), was calculated as follows ICC_1_ = (σ_ID_/(σ_ID_+σ_ID, Day_)); and ICC_2_, which included the measurement error (σ_Run_) at the denominator, was calculated as follows ICC_2_ = (σ_ID_/(σ_ID_+σ_ID, Day_+σ_Run_)).

In unadjusted models (Table 3), the between-individual variability (σ_ID_) varied between 0.15 (*APC* and *p16*) and 23.18 (*IFNγ*). The within-individual variability between Day 1 and 4 (σ_ID, Day_) varied between 0.20 (*Alu*) and 11.18 (*IFNγ*). Some of the markers (i.e., *RASFF1A* and *ET-1*) had higher between- that within-individual variability. Other markers (i.e., *IL-6* and *iNOS*) had higher within- than between-individual variability.

**Figure 1:**
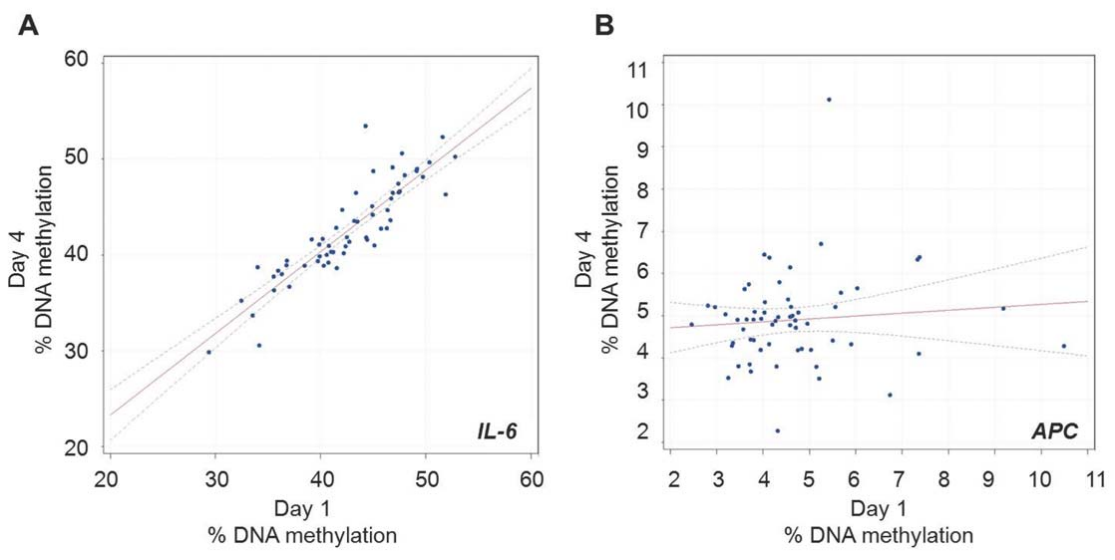
Representative scatter plots (Day 1 vs. Day 4 blood DNA methylation measures) of the biomarkers with highest (*IL-6*, panel A) and lowest (*APC*, panel B) intra-class correlation coefficients.

In unadjusted models that estimated ICCs subtracted of the pyrosequencing measurement error (ICC_1_, see Table 3), *IL-6* was the marker that showed the most stable methylation levels between Day 1 and 4 [ICC_1_ = 0.89]. *APC* was the marker that showed the highest variation between the two time points [ICC_1_ = 0.10]. Representative scatter plots for these two genes are shown in Figure 1a (*IL-6*) and 1b (*APC*), respectively. ICCs estimated without subtracting the pyrosequencing error (ICC_2_) were only moderately lower than ICC_1_ (Table 3). In order to adjust for the potential effects of PM_10_ exposure, as well as potential confounding by age, current smoking and changes in percent granulocytes in the blood counts, we also used models fitting those covariates as independent variables to calculate adjusted σ_ID_, σ_ID, Day_, and ICCs. Results from adjusted models were remarkably similar to those from unadjusted models (Table 3). We also performed additional set of analyses based on individual CpGs with both unadjusted (Table S5a) and adjusted models (Table S5b). The analyses on individual CpGs generally showed similar stability across the CpG sites within in the same gene (Table S4). However, in *hTERT* the CpG at position 3 appeared moderately more stable (adjusted ICC_1_ = 0.57) than the CpGs at position 1 (adjusted ICC_1_ = 0.15) or 2 (adjusted ICC_1_ = 0.30). Also, in *p16* the CpG at position 7 showed higher stability (adjusted ICC_1_ = 0.51) than the CpGs at positions 1–6 (adjusted ICC_1_ between 0.00 and 0.24).

In order to understand the sole effect of PM_10_ level in changing DNA methylation during Day 1and Day 4, we examined the association between exposed PM levels with DNA methylation in each gene (Table S6 and S7). We also examined whether PM levels were associated with increased or decreased percent granulocytes, monocytes, or lymphocytes during these time points (Table S8). Although DNA methylation of some of the genes was associated with PM_10_, the variance of DNA methylation was not influenced by the level of PM_10_, as shown by the adjusted models in Table 3; nor the level of PM_10_ was associated with the percent of granulocytes, monocytes, or lymphocytes.

### Marker and Genomic Characteristics Associated with DNA Methylation Stability

**Figure 2:**
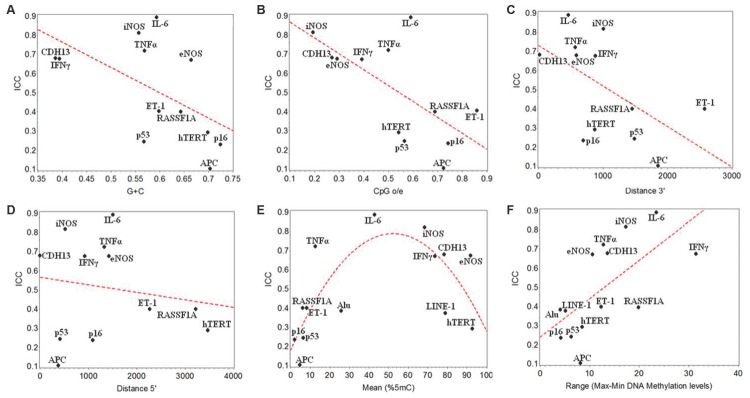
Correlations of intraclass correlation coefficients (ICCs) with DNA methylation levels and genomic characteristics of the sequences analyzed. The panels show correlations of ICCs for each of the methylation biomarkers with content of guanosine and cytosine (G+C, panel A); ratio of observed/expected CpG dinucleotides (CpG o/e; panel B); distance of repeat elements from 3′ (panel C); distance of repeat elements from 5′ (panel D); DNA methylation mean on Day 1 (panel E); range of DNA methylation on Day 1 (panel F). The scatter plots use ICC values subtracted of pyrosequencing measurement errors (ICC_1_) and estimated from models adjusted by PM_10_ exposure levels, age, current smoking, and percent blood granulocytes. Each data point corresponds to the ICC_1_ value for one biomarker, as indicated by the corresponding label.

Figure 2 shows the correlations of ICCs with the marker and genomic characteristics that we considered. In these analyses, we used ICC_1_ values from adjusted models. To evaluate the correlation of DNA methylation stability with CpG density, we examined the GC content (G+C), as well as the ratio of observed-to-expected [CpG(o/e)] within ±200bp from the first CpG site analyzed. Both these measures showed negative correlation with ICC (−0.13 estimated change in ICC_1_ [95% CI −0.27; 0.01] per a 0.1 increase in G+C, Figure 2a; and −0.08 estimated change in ICC_1_ [95% CI −0.15; −0.02 per a 0.1 increase in CpG(o/e), Figure 2b). These findings suggest that CpG-rich regions had lower stability. The distance of repeat elements in 3′ showed a moderate negative correlation with the ICC values (−0.20 estimated change in ICC_1_ [95% CI −0.40; 0.006] per each 1,000 nucleotides, Figure 2c). The distance of repeat elements in 5′ did not show correlation with ICC (−0.04 estimated change in ICC_1_ [95% CI −0.2; 0.1] per each 1,000 nucleotides, Figure 2d). The mean methylation of the markers on Day 1 did not show a linear correlation with ICCs (Figure 2e). However, visual inspection showed that the highest variations between Day 1 and 4 were found in markers with either high or low mean DNA methylation. By fitting a quadratic term regression, we found an inverted U-shaped relation between mean DNA methylation and ICCs (0.2 [95% CI 0.1; 0.4] for the linear term; −2*10^−3^, 95% CI −4*10^−3^; −1*10^−3^ for the quadratic term; both estimating the change in ICC_1_ per 10% methylcytosine units of DNA methylation). The observed range of methylation on Day 1 was positively correlated with ICC (0.2 estimated change in ICC_1_ [95% CI 0.1; 0.3] per 10% methyl-cytosine units of DNA methylation, Figure 2f).

## Discussion

In the present analysis of a set of DNA methylation markers, we identified large marker-dependent differences in the short-term variability of blood DNA methylation levels. We demonstrated that at least some of these differences can be predicted using characteristics of the nearby sequence – such as those describing CpG density – as well as based on the mean and range of methylation of the markers on Day 1. These results, albeit limited to a small-sized population with a specific condition of exposure to PM, provide a first set of information to identify methylation markers with high short-term variability.

Based on early findings in cancer tissues that showed frequent alterations in DNA cytosine methylation within promoter CpG islands, CpG-rich regions have been a primary focus of functional epigenetic studies [28], [29]. However, recent data by Irizarry et al., suggesting that a majority of functional methylation sites reside in regions less dense in CpGs termed CpG-island shores, have directed DNA methylation studies toward sequences with lower methylation density [10]. Because CpG-island shores have been shown to constitute a large proportion of tissue-specific and cancer-related methylation patterns [10], these sequences with lower methylation density are now widely considered hot spots for DNA methylation changes. In the present study based on DNA methylation measures on blood leukocyte DNA, we found a negative association between CpG nucleotide density and ICCs. This finding indicates that DNA methylation in CpG-rich areas is highly variable, whereas CpG-poor regions have more stable methylation – at least over the short time period (i.e., three days) evaluated. Key differences of our study compared with previous data on CpG-island shores [10], [30] are in the type of DNA source and the study design that we used. Our analysis was specifically conducted to draw inference for human studies using unfractionated blood leukocytes as the DNA source for methylation analyses. As we collected blood leukocyte DNA twice from each of the study participants, we could directly differentiate the amount of inter-individual and within-individual variability in the DNA methylation markers. However, evidence from previous functional studies also supports our findings. DNA methyltransferases (DNMTs), the best-characterized enzymes responsible for DNA methylation changes in somatic human tissues, have been shown to bind to specific genomic regions [31]. Recruiting of *de-novo* DNMTs is preferential to CpG-rich sequences, such as CpG islands [32], and could account for the higher short-term variation of DNA methylation that we observed in CpG-rich sequences.

One interesting observation in the present study is the finding of a marginal negative association between ICCs and the 3′ distance of repeat elements from the methylation sites analyzed. Repeat elements may spread DNA methylation to adjacent genes [17]. Paradoxically, repeat elements have also been shown to serve as insulators, protecting against *de-novo* DNA methylation [33]. In our data, methylation markers closer to repeat elements were more stable in time, suggesting that repeat elements may protect DNA methylation of neighboring genes from short-term variation. Previous investigations suggest that this finding may result from interplays involving CCCTC-binding factor (CTCF), specificity Protein 1 (SP1) and DNMTs. Repeat elements tend to be associated with heterochromatin states and inactive histone modifications, and are regarded as insulators that act like a barrier against the influences of neighboring cis-acting elements [34], [35]. This molecular property of repeat elements might underlie the high-stability of DNA methylation markers located within a short distance of a repeat element.

In our analysis, we found that the markers with mean methylation values close to 50% on Day 1 had the highest stability whereas those close to 0% or 100% showed less correlation between the two time points. We also found that the range of DNA methylation observed on Day 1 was positively correlated with the ICC values. As the markers with methylation values close to 50% were also the ones with the largest ranges (compare Figure 2e and 2f), this second finding is to be considered closely related with the inverse U-shaped correlation observed between mean DNA methylation and ICC. We suggest potential ways through which DNA methylation showed levels around 50%. The most obvious way is that methylation is derived from a variety of cell types in blood leukocytes, and the mix of different methylation patterns in each cell might determine the average leucocyte methylation values. Whereas the mix of cell types may determine differences in DNA methylation, our results of DNA methylation stability were adjusted for the number and also the proportion of different blood cell types. Another possibility is that the two alleles present in each cell could be differentially methylated, i.e. one of them methylated and the other unmethylated. However, although this is the usual pattern found in imprinted genes, allele-specific methylation is highly infrequent in non-imprinted genes. None of the markers analyzed is known to be imprinted. However, as we did not perform allele-specific methylation analysis, we cannot exclude differential allelic methylation based on our data. It is worth noting that, whereas we computed ICCs net of pyrosequencing measurement error, we did not run duplicates for bisulfite treatment and PCR-amplification, which are other potential unmeasured sources of measurement error. The association between larger methylation ranges and ICCs may in part result from the influence of residual measurement error, which is comparatively larger for the biomarkers with smaller ranges.

We studied a group of individuals with well-characterized environmental exposure to metal-rich air particles. In previous analyses, we have shown in this group exposure-dependent alterations of epigenetic and molecular markers related with pro-inflammatory and oxidative properties of the exposure, including DNA methylation [26], [36], histone modifications [36], miRNAs [37], telomere length [38], and mitochondrial DNA abundance [27]. However, in the present study we found similar ICCs in models with or without adjustment for PM_10_ levels. This finding indicates that our estimates of the stability of DNA methylation between Day 1 and 4 are independent from the levels of exposure in the steel factory. The levels of individual PM_10_ exposure in this study group ranged from high to low, and individuals with lower exposures had PM_10_ levels similar to those found outdoors in metropolitan areas in North America and Europe [26]. Therefore, our results may extend to populations of individuals living in more common environmental conditions.

We recognize several limitations to our investigation. Our results are based on a limited selection of methylation markers, which were available for the present analyses from previous and ongoing studies on the effects of metal-rich particles on DNA methylation. Our selection included markers related to inflammation, oxidative stress and cell-cycle control. Our results do not necessarily apply to other markers. Nonetheless, the markers analyzed included a variety of different genomic characteristics, as well as a wide range of methylation levels, that allowed for effectively testing the correlation of ICC with differences in CpG density, distances from repeat elements, and DNA methylation values on Day 1. Our marker selection is particularly limited if compared with the high numbers of methylation sites that can be analyzed using methylation arrays or other genome-scale approaches. However, bisulfite-PCR-pyrosequencing is the gold standard for DNA methylation analyses in short (up to 80–100 bp) sequences and provides measures that are considerably more accurate and quantitative than microarray measures [39], [40]. The higher precision of pyrosequencing-based analyses is a critical strength in the context of the statistical analyses presented in this report. We recognize the small sample size as an additional constraint that limited the precision of our statistical estimates. Our statistical strategy, however, took advantage of some of the strengths of the study, including the measurement of all methylation markers in duplicates and the availability of differential blood counts. We computed ICCs net of pyrosequencing error, we adjusted all results for percent granulocytes, and we conducted sensitivity analyses that demonstrated that the results were not dependent on changes in the proportion of other major leukocyte cell types, i.e. granulocytes, monocytes and lymphocytes (Table S2). However, some of the changes in DNA methylation between Day 1 and 4 might have been determined by proliferation of cell subpopulations within each of the major cell types (e.g., lymphocytic subpopulations). Whether short-term DNA methylation modifications in blood leukocytes are determined by actual modifications of DNA methylation or rather by clonal expansion of a subpopulation with distinctive methylation profiles remains to be determined. Finally, we only examined two time points within the same week. It is worth noting that our findings may not apply to slower progressive changes in DNA methylation, such as those associated with aging. Studies with a larger number of time points over a longer time period may provide more detailed and accurate information on cumulative long-term changes in DNA methylation.

In conclusion, we showed that DNA methylation markers in blood DNA have different degrees of short-term variability. The amount of variability in the methylation markers evaluated could be predicted based on easy-to-obtain sequence information, such as CpG density and distance from repeat element, as well as on the average and range of methylation at the first measurement. Our results provide information on short-term variability of methylation measures on blood leukocyte DNA, which are extensively used in human studies. Whether these results can be extended to other cell types remains to be determined in further investigations**.**

## Supporting Information

Figure S1 **Correlations of logit transformed ICCs with genomic characteristics of the sequences analyzed.** The panels show correlations of logit transformed ICCs for each of the methylation biomarkers with content of guanosine and cytosine (G+C, panel A); ratio of observed/expected CpG dinucleotides (CpG o/e; panel B); distance of repeat elements from 3′ (panel C); distance of repeat elements from 5′ (panel D); DNA methylation mean on Day 1 (panel E); range of DNA methylation on Day 1 (panel F). The scatter plots use ICC values subtracted of pyrosequencing measurement errors (ICC_1_) and estimated from models adjusted by PM_10_ exposure levels, age, current smoking, and percent blood granulocytes. Each data point corresponds to the ICC_1_ value for one biomarker, as indicated by the corresponding label.
(DOC)

Table S1 **PCR primer sequences.**
(DOC)

Table S2 Table S2a. Variance components and ICCs estimating the concordance between Day 1 and Day 4 DNA methylation measures. Models adjusted by PM_10_ exposure levels, age current smoking, and percent blood granulocytes. Table S2b. Variance components and ICCs estimating the concordance between Day 1 and Day 4 DNA methylation measures. Models adjusted by PM_10_ exposure levels, age current smoking, and percent blood monocytes. Annotation: σ_ID_ represents the between-subject variance in DNA methylation; σ_ID, Day_ represents the variance due to within-subject changes in DNA methylation between Day 1 and Day 4; σ_Run_ represents the variance between duplicate pyrosequencing runs on the same sample (i.e., analytical measurement error from pyrosequencing). Two types of Intraclass Correlation Coefficients (ICCs) were computed using the quantities above: ICC_1_, subtracted of the measurement error (σ_Run_), was calculated as follows ICC_1_ = (σ_ID_/(σ_ID_+σ_ID, Day_)); and ICC_2_, which included the measurement error (σ_Run_) at the denominator, was calculated as follows ICC_2_ = (σ_ID_/(σ_ID_+σ_ID, Day_+σ_Run_)). Table S2c. Variance components and ICCs estimating the concordance between Day 1 and Day 4 DNA methylation measures. Models adjusted by PM_10_ exposure levels, age current smoking, and percent blood lymphocytes. Annotation: σ_ID_ represents the between-subject variance in DNA methylation; σ_ID, Day_ represents the variance due to within-subject changes in DNA methylation between Day 1 and Day 4; σ_Run_ represents the variance between duplicate pyrosequencing runs on the same sample (i.e., analytical measurement error from pyrosequencing). Two types of Intraclass Correlation Coefficients (ICCs) were computed using the quantities above: ICC_1_, subtracted of the measurement error (σ_Run_), was calculated as follows ICC_1_ = (σ_ID_/(σ_ID_+σ_ID, Day_)); and ICC_2_, which included the measurement error (σ_Run_) at the denominator, was calculated as follows ICC_2_ = (σ_ID_/(σ_ID_+σ_ID, Day_+σ_Run_)).
(DOC)

Table S3 **Non-linear relationships between logit transformed ICCs and each of the marker characteristics.**
(DOC)

Table S4 **DNA methylation levels (%mC) at individual CpGs in Day 1 and Day 4 blood samples.**
(DOC)

Table S5 Table S5a. Variance components and ICCs based on methylation values at individual CpGs estimating the concordance between Day 1 and Day 4 DNA methylation measures. Unadjusted Models. Annotation: σ_ID_ represents the between-subject variance in DNA methylation; σ_ID, Day_ represents the variance due to within-subject changes in DNA methylation between Day 1 and Day 4*;* σ_Run_ represents the variance between duplicate pyrosequencing runs on the same sample (i.e., analytical measurement error from pyrosequencing). Two types of Intraclass Correlation Coefficients (ICCs) were computed using the quantities above: ICC_1_, subtracted of the measurement error (σ_Run_), was calculated as follows ICC_1_ = (σ_ID_/(σ_ID_+σ_ID, Day_)); and ICC_2_, which included the measurement error (σ_Run_) at the denominator, was calculated as follows ICC_2_ = (σ_ID_/(σ_ID_+σ_ID, Day_+σ_Run_)). Table S5b. Variance components and ICCs based on individual CpGs estimating the concordance between Day 1 and Day 4 DNA methylation measures. Models adjusted by PM_10_ exposure levels, age current smoking, and percent blood granulocytes. Annotation: σ_ID_ represents the between-subject variance in DNA methylation; σ_ID, Day_ represents the variance due to within-subject changes in DNA methylation between Day 1 and Day 4*;* σ_Run_ represents the variance between duplicate pyrosequencing runs on the same sample (i.e., analytical measurement error from pyrosequencing). Two types of Intraclass Correlation Coefficients (ICCs) were computed using the quantities above: ICC_1_, subtracted of the measurement error (σ_Run_), was calculated as follows ICC_1_ = (σ_ID_/(σ_ID_+σ_ID, Day_)); and ICC_2_, which included the measurement error (σ_Run_) at the denominator, was calculated as follows ICC_2_ = (σ_ID_/(σ_ID_+σ_ID, Day_+σ_Run_)).
(DOC)

Table S6 **The effect of PM_10_ level on the changes of DNA methylation between Day 1 and Day 4.**
(DOC)

Table S7 **Variance components and ICCs estimating the concordance between Day 1 and Day 4 DNA methylation measures.** Models adjusted by PM_10_ exposure + Interaction PM_10_*DAY Annotation: σ_ID_ represents the between-subject variance in DNA methylation; σ_ID, Day_ represents the variance due to within-subject changes in DNA methylation between Day 1 and Day 4*;* σ_Run_ represents the variance between duplicate pyrosequencing runs on the same sample (i.e., analytical measurement error from pyrosequencing). Two types of Intraclass Correlation Coefficients (ICCs) were computed using the quantities above: ICC_1_, subtracted of the measurement error (σ_Run_), was calculated as follows ICC_1_ = (σ_ID_/(σ_ID_+σ_ID, Day_)); and ICC_2_, which included the measurement error (σ_Run_) at the denominator, was calculated as follows ICC_2_ = (σ_ID_/(σ_ID_+σ_ID, Day_+σ_Run_)).
(DOC)

Table S8 **Association of PM_10_ levels with changes in blood cell types between Day 1 and Day 4.**
(DOC)
