# Supplementary material for: Temporal Stability of Epigenetic Markers: Sequence Characteristics and Predictors of Short-Term DNA Methylation Variations
Source: PLoS One. 2012 Jun 20;7(6):e39220. doi: 10.1371/journal.pone.0039220 (PMC3379987; doi:10.1371/journal.pone.0039220)
Supplement: Figure S1 — Correlations of logit transformed ICCs with genomic characteristics of the sequences analyzed. The panels show correlations of logit transformed ICCs for each of the methylation biomarkers with content of guanosine and cytosine (G+C, panel A); ratio of observed/expected CpG dinucleotides (CpG o/e; panel B); distance of repeat elements from 3′ (panel C); distance of repeat elements from 5′ (panel D); DNA methylation mean on Day 1 (panel E); range of DNA methylation on Day 1 (panel F). The scatter plots use ICC values subtracted of pyrosequencing measurement errors (ICC1) and estimated from models adjusted by PM10 exposure levels, age, current smoking, and percent blood granulocytes. Each data point corresponds to the ICC1 value for one biomarker, as indicated by the corresponding label. (DOC) [file pone.0039220.s001.doc]

Figure S1. Correlations of logit transformed ICCs with genomic characteristics of the sequences analyzed.


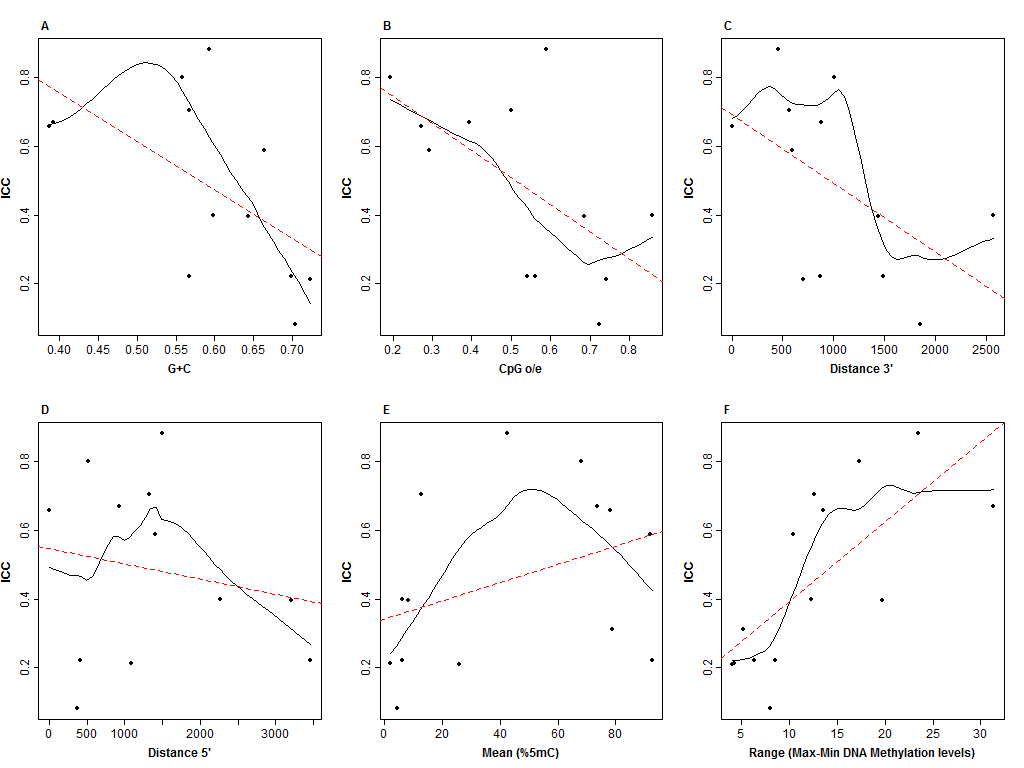


The panels show correlations of logit transformed ICCs for each of the methylation biomarkers with content of guanosine and cytosine (G+C, panel A); ratio of observed/expected CpG dinucleotides (CpG o/e; panel B); distance of repeat elements from 3’ (panel C); distance of repeat elements from 5’ (panel D); DNA methylation mean on Day 1 (panel E); range of DNA methylation on Day 1 (panel F). The scatter plots use ICC values subtracted of pyrosequencing measurement errors (ICC_1_) and estimated from models adjusted by PM_10_ exposure levels, age, current smoking, and percent blood granulocytes. Each data point corresponds to the ICC_1_ value for one biomarker, as indicated by the corresponding label.
