# Supplementary material for: Temporal Stability of Epigenetic Markers: Sequence Characteristics and Predictors of Short-Term DNA Methylation Variations
Source: PLoS One. 2012 Jun 20;7(6):e39220. doi: 10.1371/journal.pone.0039220 (PMC3379987; doi:10.1371/journal.pone.0039220)
Supplement: Table S1 — PCR primer sequences. (DOC) [file pone.0039220.s002.doc]

Table S1: PCR primer sequences

| Sequence ID | Primer | Sequence |
| --- | --- | --- |
| *APC* | Forward Primer (5’ to 3’) | TTTTGTTTGTTGGGGATTG |
|  | Reverse Primer (5’ to 3’) | Biotin -CTCCAACACCTACCCCATTT |
|  | Sequencing Primer (5’ to 3’) | GGGGTTTTGTGTTTTA |
|  | Sequence analyzed (5’ to 3’) | TTGC/TGGAGTGC/TGGGTC/TGGGAAGC/TGGAG |
| *p53* | Forward Primer (5’ to 3’) | Biotin -TTAGGAGTTTATTTAATTTAGGGAAG |
|  | Reverse Primer (5’ to 3’) | TATCCAACTTTATACCAAAAACCTC |
|  | Sequencing Primer (5’ to 3’) | TCCAAAAAACAAATAACTACTAAACTC |
|  | Sequence analyzed (5’ to 3’) | CG/AAAAACACTTTACG/ATTCG/AAACTAAAAACG/ATACTTT |
| *p16* | Forward Primer (5’ to 3’) | AGGGGTTGGTTGGTTATTAG |
|  | Reverse Primer (5’ to 3’) | Biotin - CTACCTACTCTCCCCCTCTC |
|  | Sequencing Primer (5’ to 3’) | GGTTGGTTATTAGAGGGT |
|  | Sequence analyzed (5’ to 3’) | GGGGC/TGGATC/TGC/TGTGC/TGTTC/TGGC/TGGTTGC/TG |
| *eNOS* | Forward Primer (5’ to 3’) | TGTAGTTTTAGGGTTTTGTTGGA |
|  | Reverse Primer (5’ to 3’) | Biotin-CCCCTATCCCATACACAAT |
|  | Sequencing Primer (5’ to 3’) | TATTAGTTTTAGTTTTTATA |
|  | Sequence analyzed (5’ to 3’) | GC/TGGAATTTAGGC/TGTTC/TGGTTTTTT |
| *ET-1* | Forward Primer (5’ to 3’) | TTGTTTGGGGTTGGAATAAAGT |
|  | Reverse Primer (5’ to 3’) | Biotin-ATCCTTCAACCCAAATACCCTTTT |
|  | Sequencing Primer (5’ to 3’) | GGTAGAGAGTTGTTTAAGT |
|  | Sequence analyzed (5’ to 3’) | AGAC/TGC/TGTTTTTGTATTTGC/TGTTAGGC/TGAA |
| *IL-6* | Forward Primer (5’ to 3’) | Biotin -TATTTTAGTTTTGAGAAAGGAGGTG |
|  | Reverse Primer (5’ to 3’) | CAATACTCTAAAACCCAACAAAAA |
|  | Sequencing Primer (5’ to 3’) | TCCTAATACAAACAACCCC |
|  | Sequence analyzed (5’ to 3’) | CG/AACCACACG/ACAAAAAC |
| *IFNγ* | Forward Primer (5’ to 3’) | Biotin -GTTTTTTGGATTTGATTAGTTTGA |
|  | Reverse Primer (5’ to 3’) | CAATAACAACCAAAAAAACCCA |
|  | Sequencing Primer (5’ to 3’) | TATAACTTATATATTTCATC |
|  | Sequence analyzed (5’ to 3’) | G/ATTTCCG/AAAAAAA |
| *iNOS* | Forward Primer (5’ to 3’) | AATGAGAGTTGTTGGGAAGTGTTT |
|  | Reverse Primer (5’ to 3’) | Biotin -CCACCAAACCCAACCAAACT |
|  | Sequencing Primer (5’ to 3’) | TAAAGGTATTTTTGTTTTAA |
|  | Sequence analyzed (5’ to 3’) | C/TGATTTTC/TGGGTTTTTTTTTATTTTG |
| *RASSF1A* | Forward Primer (5’ to 3’) | TTAGTGGGTAGGTTAAGTGTGTTG |
|  | Reverse Primer (5’ to 3’) | Biotin -TACCCTTCCTTCCCTCCTTC |
|  | Sequencing Primer (5’ to 3’) | AAAGTTGGTTTTTAGAAATA |
|  | Sequence analyzed (5’ to 3’) | C/TGGGTATTTTC/TGC/TGTGGTGTTTTGC/TGGTC/TGTC/TGTC/TGTTGTG |
| *CDH13* | Forward Primer (5’ to 3’) | Biotin -ATGGAATATTTTATGATGATTAAG |
|  | Reverse Primer (5’ to 3’) | CCACAAATTAATTTTACCTACATT |
|  | Sequencing Primer (5’ to 3’) | TCTCCTATTTAACTTCTTCA |
|  | Sequence analyzed (5’ to 3’) | TTCA/GACATTACATACCACA/GAAATT |
| *TNFα* | Forward Primer (5’ to 3’) | Biotin -TGAGGGGTATTTTTGATGTTTGT |
|  | Reverse Primer (5’ to 3’) | CCAACAACTACCTTTATATATCCC |
|  | Sequencing Primer (5’ to 3’) | ATAAACCCTACACCTTCTAT |
|  | Sequence analyzed (5’ to 3’) | CTCG/AATTTCTTCTCCATCG/ACG/AAAAACG/AAAAATTT |
| *hTERT* | Forward Primer (5’ to 3’) | AGGTTTTGGATGTTAGGGATTTT |
|  | Reverse Primer (5’ to 3’) | Biotin -CCACAAAACCCTAAAACTTCTCC |
|  | Sequencing Primer (5’ to 3’) | GGAGTTGTTTGGGAAT |
|  | Sequence analyzed (5’ to 3’) | TAC/TGC/TGTAGTGTTTTTAC/TGGGGTGTTTTTT |
| *Alu* | Forward Primer (5’ to 3’) | Biotin-TTTTTATTAAAAATATAAAAATT |
|  | Reverse Primer (5’ to 3’) | CCCAAACTAAAATACAATAA |
|  | Sequencing Primer (5’ to 3’) | AATAACTAAAATTACAAAC |
|  | Sequence analyzed (5’ to 3’) | G/AC/TG/AC/TG/ACCACCA |
| LINE-1 | Forward Primer (5’ to 3’) | TTTTGAGTTAGGTGTGGGATATA |
|  | Reverse Primer (5’ to 3’) | Biotin-AAAATCAAAAAATTCCCTTTC |
|  | Sequencing Primer (5’ to 3’) | AGTTAGGTGTGGGATATAGT |
|  | Sequence analyzed (5’ to 3’) | TTC/TGTGGTGC/TGTC/TG |
