# Supplementary material for: Temporal Stability of Epigenetic Markers: Sequence Characteristics and Predictors of Short-Term DNA Methylation Variations
Source: PLoS One. 2012 Jun 20;7(6):e39220. doi: 10.1371/journal.pone.0039220 (PMC3379987; doi:10.1371/journal.pone.0039220)
Supplement: Table S2 — a. Variance components and ICCs estimating the concordance between Day 1 and Day 4 DNA methylation measures. Models adjusted by PM10 exposure levels, age current smoking, and percent blood granulocytes. Table S2b. Variance components and ICCs estimating the concordance between Day 1 and Day 4 DNA methylation measures. Models adjusted by PM10 exposure levels, age current smoking, and percent blood monocytes. Annotation: σID represents the between-subject variance in DNA methylation; σID, Day represents the variance due to within-subject changes in DNA methylation between Day 1 and Day 4; σRun represents the variance between duplicate pyrosequencing runs on the same sample (i.e., analytical measurement error from pyrosequencing). Two types of Intraclass Correlation Coefficients (ICCs) were computed using the quantities above: ICC1, subtracted of the measurement error (σRun), was calculated as follows ICC1 = (σID/(σID+σID, Day)); and ICC2, which included the measurement error (σRun) at the denominator, was calculated as follows ICC2 = (σID/(σID+σID, Day+σRun)). Table S2c. Variance components and ICCs estimating the concordance between Day 1 and Day 4 DNA methylation measures. Models adjusted by PM10 exposure levels, age current smoking, and percent blood lymphocytes. Annotation: σID represents the between-subject variance in DNA methylation; σID, Day represents the variance due to within-subject changes in DNA methylation between Day 1 and Day 4; σRun represents the variance between duplicate pyrosequencing runs on the same sample (i.e., analytical measurement error from pyrosequencing). Two types of Intraclass Correlation Coefficients (ICCs) were computed using the quantities above: ICC1, subtracted of the measurement error (σRun), was calculated as follows ICC1 = (σID/(σID+σID, Day)); and ICC2, which included the measurement error (σRun) at the denominator, was calculated as follows ICC2 = (σID/(σID+σID, Day+σRun)). (DOC) [file pone.0039220.s003.doc]

Table S2a. Variance components and ICCs estimating the concordance between Day 1 and Day 4 DNA methylation measures. Models adjusted by PM_10_ exposure levels, age current smoking, and percent blood **granulocytes.**

| **Marker** | **σ_ID_** | **σ_ID, Day_** | **σ_Run_** | **ICC_1_** | **ICC_2_** |
| --- | --- | --- | --- | --- | --- |
| *APC* | 0.14 | 1.29 | 0.36 | 0.10 | 0.08 |
| *CDH13* | 4.72 | 2.26 | 0.25 | 0.68 | 0.65 |
| *eNOS* | 2.69 | 1.35 | 0.60 | 0.67 | 0.58 |
| *ET-1* | 5.37 | 7.33 | 0.13 | 0.42 | 0.42 |
| *hTERT* | 0.54 | 1.09 | 0.54 | 0.33 | 0.25 |
| *IFNγ* | 18.27 | 9.94 | 0.23 | 0.65 | 0.64 |
| *IL6* | 22.74 | 2.65 | 0.30 | 0.90 | 0.89 |
| *iNOS* | 11.53 | 2.54 | 0.30 | 0.82 | 0.80 |
| *p16* | 0.16 | 0.49 | 0.07 | 0.25 | 0.23 |
| *p53* | 0.58 | 1.64 | 0.23 | 0.26 | 0.24 |
| *RASSF1A* | 8.15 | 11.00 | 0.15 | 0.43 | 0.42 |
| *TNFα* | 3.67 | 1.70 | 0.17 | 0.68 | 0.66 |
| *Alu* | 0.11 | 0.20 | 0.27 | 0.37 | 0.20 |
| LINE-1 | 0.59 | 0.96 | 0.33 | 0.38 | 0.31 |

Annotation: σ_ID_ represents the between-subject variance in DNA methylation; σ_ID, Day_ represents the variance due to within-subject changes in DNA methylation between Day 1 and Day 4*;* σ_Run_ represents the variance between duplicate pyrosequencing runs on the same sample (i.e., analytical measurement error from pyrosequencing). Two types of Intraclass Correlation Coefficients (ICCs) were computed using the quantities above: ICC_1_, subtracted of the measurement error (σ_Run_), was calculated as follows ICC_1_=(σ_ID_ / (σ_ID_ + σ_ID, Day_)); and ICC_2_, which included the measurement error (σ_Run_) at the denominator, was calculated as follows ICC_2_=(σ_ID_ / (σ_ID_ + σ_ID, Day_ + σ_Run_)).

Table S2b. Variance components and ICCs estimating the concordance between Day 1 and Day 4 DNA methylation measures. Models adjusted by PM_10_ exposure levels, age current smoking, and percent **blood monocytes**.

| **Marker** | **σ_ID_** | **σ_ID, Day_** | **σ_Run_** | **ICC_1_** | **ICC_2_** |
| --- | --- | --- | --- | --- | --- |
| *APC* | 0.13 | 1.28 | 0.36 | 0.09 | 0.08 |
| *CDH13* | 4.85 | 2.03 | 0.25 | 0.71 | 0.68 |
| *eNOS* | 2.39 | 1.42 | 0.60 | 0.63 | 0.54 |
| *ET-1* | 5.17 | 7.41 | 0.13 | 0.41 | 0.41 |
| *hTERT* | 0.51 | 1.12 | 0.53 | 0.31 | 0.24 |
| *IFNγ* | 17.45 | 10.44 | 0.23 | 0.63 | 0.62 |
| *IL6* | 23.22 | 2.70 | 0.30 | 0.90 | 0.89 |
| *iNOS* | 11.56 | 2.40 | 0.30 | 0.83 | 0.81 |
| *p16* | 0.17 | 0.50 | 0.07 | 0.25 | 0.23 |
| *p53* | 0.58 | 1.66 | 0.23 | 0.26 | 0.24 |
| *RASSF1A* | 7.65 | 11.39 | 0.15 | 0.40 | 0.40 |
| *TNFα* | 3.62 | 1.71 | 0.17 | 0.68 | 0.66 |
| *Alu* | 0.08 | 0.19 | 0.27 | 0.31 | 0.15 |
| LINE-1 | 0.58 | 0.99 | 0.33 | 0.37 | 0.30 |

Annotation: σ_ID_ represents the between-subject variance in DNA methylation; σ_ID, Day_ represents the variance due to within-subject changes in DNA methylation between Day 1 and Day 4*;* σ_Run_ represents the variance between duplicate pyrosequencing runs on the same sample (i.e., analytical measurement error from pyrosequencing). Two types of Intraclass Correlation Coefficients (ICCs) were computed using the quantities above: ICC_1_, subtracted of the measurement error (σ_Run_), was calculated as follows ICC_1_=(σ_ID_ / (σ_ID_ + σ_ID, Day_)); and ICC_2_, which included the measurement error (σ_Run_) at the denominator, was calculated as follows ICC_2_=(σ_ID_ / (σ_ID_ + σ_ID, Day_ + σ_Run_)).

Table S2c. Variance components and ICCs estimating the concordance between Day 1 and Day 4 DNA methylation measures. Models adjusted by PM_10_ exposure levels, age current smoking, and percent **blood lymphocytes**.

| **Marker** | **σ_ID_** | **σ_ID, Day_** | **σ_Run_** | **ICC_1_** | **ICC_2_** |
| --- | --- | --- | --- | --- | --- |
| *APC* | 0.13 | 1.28 | 0.36 | 0.09 | 0.07 |
| *CDH13* | 4.94 | 2.39 | 0.25 | 0.67 | 0.65 |
| *eNOS* | 2.55 | 1.37 | 0.60 | 0.65 | 0.56 |
| *ET-1* | 5.20 | 7.39 | 0.13 | 0.41 | 0.41 |
| *hTERT* | 0.52 | 1.11 | 0.53 | 0.32 | 0.24 |
| *IFNγ* | 20.10 | 11.21 | 0.23 | 0.64 | 0.64 |
| *IL6* | 23.01 | 2.60 | 0.30 | 0.90 | 0.89 |
| *iNOS* | 11.80 | 2.51 | 0.30 | 0.82 | 0.81 |
| *p16* | 0.17 | 0.49 | 0.07 | 0.25 | 0.23 |
| *p53* | 0.57 | 1.66 | 0.23 | 0.25 | 0.23 |
| *RASSF1A* | 7.67 | 11.19 | 0.15 | 0.41 | 0.40 |
| *TNFα* | 3.67 | 1.70 | 0.17 | 0.68 | 0.66 |
| *Alu* | 0.12 | 0.19 | 0.27 | 0.39 | 0.21 |
| LINE-1 | 0.59 | 0.97 | 0.33 | 0.38 | 0.31 |

Annotation: σ_ID_ represents the between-subject variance in DNA methylation; σ_ID, Day_ represents the variance due to within-subject changes in DNA methylation between Day 1 and Day 4*;* σ_Run_ represents the variance between duplicate pyrosequencing runs on the same sample (i.e., analytical measurement error from pyrosequencing). Two types of Intraclass Correlation Coefficients (ICCs) were computed using the quantities above: ICC_1_, subtracted of the measurement error (σ_Run_), was calculated as follows ICC_1_=(σ_ID_ / (σ_ID_ + σ_ID, Day_)); and ICC_2_, which included the measurement error (σ_Run_) at the denominator, was calculated as follows ICC_2_=(σ_ID_ / (σ_ID_ + σ_ID, Day_ + σ_Run_)).
