# Supplementary material for: Temporal Stability of Epigenetic Markers: Sequence Characteristics and Predictors of Short-Term DNA Methylation Variations
Source: PLoS One. 2012 Jun 20;7(6):e39220. doi: 10.1371/journal.pone.0039220 (PMC3379987; doi:10.1371/journal.pone.0039220)
Supplement: Table S3 — Non-linear relationships between logit transformed ICCs and each of the marker characteristics. (DOC) [file pone.0039220.s004.doc]

Table S3. Non-linear relationships between logit transformed ICCs and each of the marker characteristics.

|  | **β*** | **SE** | **(95% CI)** | | **P-value** |
| --- | --- | --- | --- | --- | --- |
| G+C | -6.25 | 3.07 | (-13.1; | 0.60) | 0.070 |
| CpG o/e | -3.46 | 1.60 | (-7.02; | 0.11) | 0.056 |
| Repeat elements: distance at 3' | -0.93 | 0.49 | (-2.03; | 0.17) | 0.089 |
| Repeat elements: distance at 5' | -0.10 | 0.36 | (-0.91; | 0.71) | 0.795 |
| Mean (%5mC) | 10.78 | 3.90 | (2.21; | 19.35) | 0.018 |
| β Coefficient for the quadratic effect | -0.10 | 0.04 | (-0.20; | -0.01) | 0.030 |
| Range (Max-Min DNA Methylation levels) | 0.11 | 0.03 | (0.04; | 0.18) | 0.006 |

* Outcome was logit-transformed
