# Supplementary material for: Temporal Stability of Epigenetic Markers: Sequence Characteristics and Predictors of Short-Term DNA Methylation Variations
Source: PLoS One. 2012 Jun 20;7(6):e39220. doi: 10.1371/journal.pone.0039220 (PMC3379987; doi:10.1371/journal.pone.0039220)
Supplement: Table S4 — DNA methylation levels (%mC) at individual CpGs in Day 1 and Day 4 blood samples. (DOC) [file pone.0039220.s005.doc]

Table S4. DNA methylation levels (%mC) at individual CpGs in Day 1 and Day 4 blood samples

|  |  | **Day 1** | | **Day 4** | | **Difference** | **(95% CI)** | | **P-value** |
| --- | --- | --- | --- | --- | --- | --- | --- | --- | --- |
| **Gene** | **Position** | **Mean** | **(SE)** | **Mean** | **(SE)** |  |  |  |  |
| *APC* | *Overall* | 4.7 | (0.13) | 4.9 | (0.13) | 0.24 | (0.04; | 0.44) | 0.02 |
|  | *Pos. 1* | 5.8 | (0.21) | 6.1 | (0.20) | 0.35 | (-0.17; | 0.88) | 0.18 |
|  | *Pos. 2* | 5.4 | (0.17) | 5.5 | (0.17) | 0.06 | (-0.39; | 0.51) | 0.79 |
|  | *Pos. 3* | 3.6 | (0.18) | 3.9 | (0.17) | 0.29 | (-0.20; | 0.78) | 0.25 |
|  | *Pos. 4* | 3.9 | (0.18) | 4.1 | (0.18) | 0.20 | (-0.29; | 0.69) | 0.42 |
| *CDH13* | *Overall* | 78.0 | (0.33) | 77.4 | (0.35) | -0.59 | (-1.13; | -0.06) | 0.03 |
|  | *Pos. 1* | 84.3 | (0.32) | 83.8 | (0.32) | -0.57 | (-1.09; | -0.05) | 0.03 |
|  | *Pos. 2* | 72.1 | (0.42) | 71.0 | (0.42) | -1.12 | (-1.82; | -0.42) | 0.00 |
| *eNOS* | *Overall* | 91.9 | (0.3) | 92 | (0.25) | 0.14 | (-0.33; | 0.6) | 0.56 |
|  | *Pos. 1* | 90.3 | (0.38) | 90.9 | (0.38) | 0.59 | (-0.19; | 1.37) | 0.14 |
|  | *Pos. 2* | 88.4 | (0.27) | 88.6 | (0.27) | 0.17 | (-0.34; | 0.69) | 0.50 |
|  | *Pos. 3* | 96.9 | (0.38) | 96.5 | (0.38) | -0.37 | (-1.19; | 0.45) | 0.37 |
| *ET-1* | *Overall* | 6.2 | (0.42) | 6.3 | (0.4) | 0.14 | (-0.37; | 0.64) | 0.59 |
|  | *Pos. 1* | 3.9 | (0.36) | 4.2 | (0.36) | 0.26 | (-0.46; | 0.99) | 0.47 |
|  | *Pos. 2* | 3.5 | (0.28) | 3.5 | (0.28) | -0.01 | (-0.61; | 0.58) | 0.96 |
|  | *Pos. 3* | 9.6 | (0.71) | 9.2 | (0.71) | -0.38 | (-2.08; | 1.31) | 0.65 |
|  | *Pos. 4* | 7.9 | (0.63) | 7.7 | (0.63) | -0.13 | (-1.68; | 1.42) | 0.87 |
| *hTERT* | *Overall* | 92.6 | (0.18) | 92.6 | (0.15) | 0.02 | (-0.32; | 0.36) | 0.89 |
|  | *Pos. 1* | 93.9 | (0.13) | 93.9 | (0.14) | -0.03 | (-0.39; | 0.33) | 0.86 |
|  | *Pos. 2* | 93.8 | (0.33) | 93.7 | (0.33) | -0.14 | (-1.00; | 0.72) | 0.74 |
|  | *Pos. 3* | 90.0 | (0.21) | 90.2 | (0.22) | 0.20 | (-0.27; | 0.67) | 0.40 |
| *IFNγ* | *Overall* | 73.8 | (0.78) | 73 | (0.73) | -0.81 | (-2.03; | 0.41) | 0.19 |
|  | *Pos. 1* | 72.5 | (0.75) | 71.8 | (0.75) | -0.75 | (-2.00; | 0.51) | 0.24 |
|  | *Pos. 2* | 75.0 | (0.74) | 74.2 | (0.74) | -0.80 | (-2.00; | 0.41) | 0.19 |
| *IL-6* | *Overall* | 42.6 | (0.65) | 42.6 | (0.62) | -0.02 | (-0.64; | 0.6) | 0.95 |
|  | *Pos. 1* | 46.3 | (0.71) | 46.7 | (0.71) | 0.40 | (-0.35; | 1.15) | 0.29 |
|  | *Pos. 2* | 38.8 | (0.67) | 38.3 | (0.67) | -0.47 | (-1.22; | 0.29) | 0.22 |
| *iNOS* | *Overall* | 68.2 | (0.46) | 67.6 | (0.48) | -0.61 | (-1.19; | -0.02) | 0.04 |
|  | *Pos. 1* | 49.5 | (0.6) | 49.1 | (0.60) | -0.39 | (-1.19; | 0.42) | 0.34 |
|  | *Pos. 2* | 87.1 | (0.5) | 86.4 | (0.5) | -0.66 | (-1.29; | -0.04) | 0.04 |
| *p16* | *Overall* | 2.2 | (0.09) | 2.3 | (0.09) | 0.15 | (0.04; | 0.27) | 0.01 |
|  | *Pos. 1* | 2.0 | (0.12) | 2.4 | (0.12) | 0.34 | (0.03; | 0.66) | 0.03 |
|  | *Pos. 2* | 2.7 | (0.14) | 2.9 | (0.14) | 0.19 | (-0.16; | 0.54) | 0.28 |
|  | *Pos. 3* | 1.6 | (0.14) | 1.9 | (0.14) | 0.29 | (-0.11; | 0.68) | 0.16 |
|  | *Pos. 4* | 2.2 | (0.09) | 2.2 | (0.09) | -0.03 | (-0.28; | 0.22) | 0.81 |
|  | *Pos. 5* | 1.8 | (0.16) | 2.0 | (0.16) | 0.13 | (-0.33; | 0.59) | 0.58 |
|  | *Pos. 6* | 1.7 | (0.12) | 1.8 | (0.12) | 0.08 | (-0.24; | 0.39) | 0.62 |
|  | *Pos. 7* | 3.1 | (0.16) | 3.3 | (0.16) | 0.22 | (-0.13; | 0.57) | 0.21 |
| *p53* | *Overall* | 6.2 | (0.17) | 6.3 | (0.17) | 0.05 | (-0.19; | 0.28) | 0.69 |
|  | *Pos. 1* | 3.0 | (0.12) | 2.9 | (0.12) | -0.13 | (-0.45; | 0.18) | 0.40 |
|  | *Pos. 2* | 12.0 | (0.39) | 11.5 | (0.39) | -0.42 | (-1.42; | 0.58) | 0.40 |
|  | *Pos. 3* | 3.6 | (0.13) | 3.6 | (0.13) | 0.00 | (-0.31; | 0.32) | 0.98 |
|  | *Pos. 4* | 7.0 | (0.27) | 6.5 | (0.27) | -0.44 | (-1.07; | 0.2) | 0.17 |
| *RASSF1A* | *Overall* | 7.5 | (0.46) | 7.1 | (0.46) | -0.41 | (-0.99; | 0.17) | 0.16 |
|  | *Pos. 1* | 3.7 | (0.25) | 3.0 | (0.25) | -0.65 | (-1.28; | -0.02) | 0.04 |
|  | *Pos. 2* | 10.2 | (0.64) | 8.5 | (0.64) | -1.71 | (-3.2; | -0.21) | 0.03 |
|  | *Pos. 3* | 8.5 | (0.72) | 7.1 | (0.72) | -1.31 | (-2.92; | 0.29) | 0.11 |
|  | *Pos. 4* | 10.7 | (0.71) | 9.1 | (0.71) | -1.67 | (-3.24; | -0.10) | 0.04 |
| *TNFα* | *Overall* | 12.8 | (0.33) | 12.5 | (0.33) | -0.27 | (-0.76; | 0.22) | 0.27 |
|  | *Pos. 1* | 7.8 | (0.31) | 7.7 | (0.3) | -0.10 | (-0.72; | 0.53) | 0.76 |
|  | *Pos. 2* | 12.5 | (0.32) | 12.1 | (0.31) | -0.42 | (-1.10; | 0.26) | 0.22 |
|  | *Pos. 3* | 13.1 | (0.37) | 13.0 | (0.37) | -0.06 | (-0.75; | 0.62) | 0.85 |
|  | *Pos. 4* | 17.8 | (0.44) | 17.1 | (0.44) | -0.66 | (-1.33; | 0.01) | 0.06 |
| *Alu* | *Overall* | 25.8 | (0.1) | 25.8 | (0.08) | -0.02 | (-0.23; | 0.19) | 0.85 |
|  | *Pos. 1* | 35.3 | (0.14) | 35.4 | (0.15) | 0.13 | (-0.24; | 0.51) | 0.48 |
|  | *Pos. 2* | 25.6 | (0.12) | 25.5 | (0.12) | -0.08 | (-0.40; | 0.24) | 0.61 |
|  | *Pos. 3* | 16.7 | (0.1) | 16.6 | (0.10) | -0.06 | (-0.3; | 0.18) | 0.63 |
| LINE-1 | *Overall* | 78.8 | (0.13) | 78.8 | (0.15) | -0.07 | (-0.35; | 0.22) | 0.65 |
|  | *Pos. 1* | 81.7 | (0.25) | 81.8 | (0.25) | 0.01 | (-0.60; | 0.62) | 0.97 |
|  | *Pos. 2* | 78.2 | (0.13) | 78.1 | (0.13) | -0.11 | (-0.40; | 0.19) | 0.47 |
|  | *Pos. 3* | 76.5 | (0.2) | 76.7 | (0.20) | 0.17 | (-0.32; | 0.66) | 0.49 |
