# Supplementary material for: Temporal Stability of Epigenetic Markers: Sequence Characteristics and Predictors of Short-Term DNA Methylation Variations
Source: PLoS One. 2012 Jun 20;7(6):e39220. doi: 10.1371/journal.pone.0039220 (PMC3379987; doi:10.1371/journal.pone.0039220)
Supplement: Table S6 — The effect of PM10 level on the changes of DNA methylation between Day 1 and Day 4. (DOC) [file pone.0039220.s007.doc]

Table S6. The effect of PM_10_ level on the changes of DNA methylation between Day 1 and Day 4

| **Gene** | **β*** | **SE** | **(95% CI)** | | **P-value** |
| --- | --- | --- | --- | --- | --- |
| *APC* | 0.31 | 0.15 | (0.00; | 0.62) | 0.048 |
| *CDH13* | -0.11 | 0.40 | (-0.92; | 0.70) | 0.795 |
| *eNOS* | -0.75 | 0.32 | (-1.39; | -0.11) | 0.023 |
| *ET-1* | -0.06 | 0.24 | (-0.55; | 0.43) | 0.802 |
| *hTERT* | -0.12 | 0.13 | (-0.38; | 0.14) | 0.368 |
| *IFNγ* | 0.65 | 0.96 | (-1.27; | 2.56) | 0.502 |
| *IL6* | 0.58 | 0.86 | (-1.13; | 2.29) | 0.501 |
| *iNOS* | -0.40 | 0.62 | (-1.63; | 0.84) | 0.526 |
| *p16* | 0.06 | 0.11 | (-0.16; | 0.28) | 0.577 |
| *p53* | 0.06 | 0.11 | (-0.16; | 0.29) | 0.580 |
| *RASSF1A* | 0.55 | 0.18 | (0.19; | 0.90) | 0.003 |
| *TNFα* | 0.03 | 0.36 | (-0.68; | 0.75) | 0.928 |
| *Alu* | -0.18 | 0.09 | (-0.36; | 0.00) | 0.044 |
| *LINE-1* | -0.27 | 0.15 | (-0.57; | 0.04) | 0.084 |

* β for an increment equal to the difference between the 90th and 10th percentile of PM_10_.
