# Supplementary material for: Temporal Stability of Epigenetic Markers: Sequence Characteristics and Predictors of Short-Term DNA Methylation Variations
Source: PLoS One. 2012 Jun 20;7(6):e39220. doi: 10.1371/journal.pone.0039220 (PMC3379987; doi:10.1371/journal.pone.0039220)
Supplement: Table S8 — Association of PM10 levels with changes in blood cell types between Day 1 and Day 4. (DOC) [file pone.0039220.s009.doc]

Table S8. Association of PM_10_ levels with changes in blood cell types between Day 1and Day 4

| **Outcome** | **β*** | **SE** | **(95% CI)** | | **P-value** |
| --- | --- | --- | --- | --- | --- |
| *Granulocyte* | 1.12 | 1.22 | (-1.32; | 3.56) | 0.361 |
| *Monocyte* | -0.01 | 0.49 | (-0.99 | 0.98) | 0.989 |
| *Lymphocytes* | -1.13 | 1.11 | (-3.34; | 1.08) | 0.310 |

* β for an increment equal to the difference between the 90^th^ and 10^th^ percentile of PM_10_.
